# Hypofunctional Dopamine Uptake and Antipsychotic Treatment-Resistant Schizophrenia

**DOI:** 10.3389/fpsyt.2019.00314

**Published:** 2019-05-28

**Authors:** Davide Amato, Anna Kruyer, Anne-Noël Samaha, Andreas Heinz

**Affiliations:** ^1^Department of Neuroscience, Medical University of South Carolina, Charleston, SC, United States; ^2^Department of Pharmacology and Physiology, Faculty of Medicine, Université de Montréal, Montreal, QC, Canada; ^3^Department of Psychiatry, Charité University Medicine Berlin, Campus Charité Mitte, Berlin, Germany

**Keywords:** schizophrenia, drug addiction, antipsychotic efficacy, antipsychotic-resistant schizophrenia, dopamine transporter, dopamine synthesis, dopamine release

## Abstract

Antipsychotic treatment resistance in schizophrenia remains a major issue in psychiatry. Nearly 30% of patients with schizophrenia do not respond to antipsychotic treatment, yet the underlying neurobiological causes are unknown. All effective antipsychotic medications are thought to achieve their efficacy by targeting the dopaminergic system. Here we review early literature describing the fundamental mechanisms of antipsychotic drug efficacy, highlighting mechanistic concepts that have persisted over time. We then reconsider the original framework for understanding antipsychotic efficacy in light of recent advances in our scientific understanding of the dopaminergic effects of antipsychotics. Based on these new insights, we describe a role for the dopamine transporter in the genesis of both antipsychotic therapeutic response and primary resistance. We believe that this discussion will help delineate the dopaminergic nature of antipsychotic treatment-resistant schizophrenia.

## Introduction

Schizophrenia is a psychiatric condition often involving a complex genetic predisposition (1–3) as well as vulnerability to certain environmental factors (4), eventually culminating in symptoms clinically defined as positive (emergent symptoms, including hallucinations and delusions) or negative (characterized by loss of a particular function, including apathy and lack of motivation) (5–7). Additionally, a proportion of patients with schizophrenia are impaired on standard neurocognitive tasks (8), and this is considered an important correlate of disease severity (9–12). The fundamental neurobiological maladaptations underlying the symptoms of schizophrenia are not completely understood. Regardless, sub-chronic blockade of a proportion (60–80%) of dopamine D_2/3_ receptors (which we will refer to as “D_2_”) is considered to underlie treatment efficacy in schizophrenia (13). Previous and recent literature supports the effectiveness of D_2_ antagonism compared to any alternative pharmacological intervention (14–17). However, blocking dopamine receptors is not an effective therapeutic mechanism for all individuals with schizophrenia (18–24). For example, some patients with first-episode psychosis do not respond to antipsychotic treatment (25). Lack of response to antipsychotic treatment can also be “acquired” and can develop over time with long-term treatment regimens (23, 26) or can develop after a period of treatment abstinence, such as that which occurs during medication nonadherence (27–30). In many of these cases, patients unresponsive to first-line antipsychotic treatments are instead responsive to clozapine (18, 31, 32). Furthermore, there exists an additional group of patients with schizophrenia who will not respond to clozapine or to any other antipsychotic drug. This category of patients is defined as “ultra-resistant” (18, 33).

Whether all instances of antipsychotic resistance share a common neurobiological mechanism is not clear (10, 24, 34–39), nor is there a precise behavioral signature indicating its clinical manifestation, since criteria to define resistance to antipsychotic treatment were standardized only recently (40). It is not within the scope of this review to contribute to the behavioral definition of treatment resistance in schizophrenia. Rather the focus here is narrowed onto the putative role of dopamine clearance in the expression of primary antipsychotic-resistant schizophrenia (i.e., patients with first episode psychosis who never responded to treatment). We do not exclude the possibility that alterations in other neurotransmitter systems might also be involved, nor do we exclude that the dopaminergic mechanisms described here will also apply to other forms of antipsychotic resistance. Simply, we focus on dopamine, because clinical observations emphasize the importance of this neurotransmitter in the pathophysiology of psychosis (41–43) and its treatment (44). Our attention on dopamine clearance is motivated by recent data from *ex vivo* and *in vivo* studies with animal models demonstrating that antipsychotic failure is accompanied by tolerance to antipsychotic-induced increases in basal dopamine and dopamine turnover, and that the dopamine transporter (DAT) is a key moderator of both extracellular dopamine and antipsychotic response (35, 38, 45). The link between preserved, or slightly elevated, dopaminergic tone and antipsychotic responsiveness has also been observed in humans with schizophrenia (46). Recent interpretations of these data suggest that a preserved extracellular dopaminergic tone might have an important pharmacological role in the therapeutic efficacy of antipsychotics (24). These observations have been directly and indirectly supported by independent studies (38, 47–50). Due to space limitations, we will only briefly outline dopaminergic biomarkers described in the literature that appear relevant to understanding antipsychotic responsiveness. We will then conclude with the suggestion that DAT could be a more powerful moderator of antipsychotic efficacy and failure than currently recognized. Changes in DAT expression and/or function alone can alter the expected response to antipsychotic medications, making DAT a highly relevant protein when considering the dopaminergic nature of antipsychotic-resistant schizophrenia.

## Dopaminergic Dysregulation in Schizophrenia

Before discussing dopaminergic mechanisms of antipsychotic efficacy, it is important to describe the dopaminergic signaling abnormalities in schizophrenia that are targeted by antipsychotic drugs. As described in the Introduction, the underlying etiology and neuropathology of schizophrenia symptoms are still unclear. Genetic studies point to associations with genes regulating neurodevelopment, the immune system, and dopaminergic and glutamatergic transmission (2, 51), while other studies demonstrate a potential role for disruption of multiple intracellular signaling pathways in schizophrenia (52). Furthermore, environmental factors linked to schizophrenia such as migration or obstetric infection can change dopamine neurotransmission (4), in addition to other neurobiological systems (53–58). Despite the many factors that appear to contribute to schizophrenia, treatment has focused on correcting a dysregulated dopaminergic system by inhibiting dopaminergic transmission. However, it should be noted that the efficacy of pharmacologically targeting the dopaminergic system in schizophrenia does not definitively prove a dopaminergic dysregulation. Dopamine has a powerful neuromodulatory role in the brain and in the basal ganglia in particular and it can regulate motor activity as well as motivation and cognition. Since all of these functions are impacted in schizophrenia, it should not be surprising that many antidopaminergic drugs are effective (or deleterious) for schizophrenia symptoms, even though the observable symptoms may have some other underlying cause(s). Thus, the dopaminergic system should be seen as a treatment pathway capable of affecting behavioral features that appear to be disrupted in schizophrenia, but that may be caused by alterations in other neurotransmitter systems.

## Mechanisms of Antipsychotic Responsiveness

Brain dopamine receptor blockade has been embraced as a mechanism for the therapeutic efficacy of antipsychotic drugs for over 60 years (9, 59). Thus, very frequently, researchers have focused on the interactions between molecule(s) and receptor(s) to describe antipsychotic mechanisms. Although this approach is correct in principle, practically it may be too simplistic. Receptors do not act in isolation. Receptors on neurons are connected* via* synapses and organized into networks within neuronal circuitries. Receptors are also functionally linked with intracellular molecular networks that control membrane excitability, as well as neurotransmitter synthesis, release, and metabolism, and by these mechanisms, neurons can regulate their own activity. Due to the nature of neural signaling, changes in the inactivation or activation of neural receptors with antipsychotic drugs, or with any other compound, which cause local intracellular changes, will affect other cell populations through signal propagation along neural pathways. Thus, antipsychotic medications can impact neurotransmitter synthesis, release, and metabolism not only in neurons that directly interact with antipsychotics but also in those neurons that are part of the same neural circuitry. Therefore, a proper understanding of the mechanisms underlying antipsychotic responsiveness should not simply describe the chemical interactions between antipsychotic drugs and their target receptors, but should consider modifications induced by antipsychotics at the cellular and circuit levels. We will focus on neuroadaptations occurring at the cellular level that link receptors to synthesis, release, and uptake of extracellular dopamine.

## Striatal D_2_ Receptor Blockade in Treatment-Responsive Schizophrenia

Striatal D_2_ receptor blockade is considered the most effective mechanism to reduce psychotic symptoms in schizophrenia (60, 61). Extra-striatal mechanisms of antipsychotics have been debated previously (62) and will not be discussed here. The general theory of the therapeutic efficacy of antipsychotics builds on two main observations. First, clinical potency of antipsychotics, including clozapine, is directly related to their affinity for the dopamine D_2_ receptor *in vitro* (14, 15). This is substantiated by evidence that therapeutic concentrations of antipsychotics in the plasma or in the spinal fluid accurately match the antipsychotic dissociation constant (K_d_) at D_2_ receptors (63). Secondly, therapeutic concentrations of all antipsychotics (typical and atypical) produce a similar D_2_ receptor occupancy (13, 59, 64). Although this observation does not strictly apply for clozapine (21) or quetiapine (65), it has been shown that D_2_ receptor occupancy in the human brain ranges between 70% and 80% within 2 h of treatment and remains elevated for over 24 h for both typical and atypical antipsychotics (21, 66, 67). D_2_ receptor occupancy with clozapine (20) and quetiapine (68, 69), on the other hand, decreases significantly within 24 h. Based on these findings, Seeman and Tallerico (63) suggested that the main difference between typical and atypical antipsychotics is the temporal decay of antipsychotic binding to the D_2_ receptor when challenged by endogenous dopamine. In fact, antipsychotics compete with endogenous dopamine within the synaptic space and the presence of dopamine would theoretically affect the concentration of antipsychotic required to reach a particular range of D_2_ receptor occupancy. Subsequently, it was observed that the dissociation rate constant, k_off_ (rather than association rate constant, k_on_), largely accounts for the difference in binding affinity when comparing typical and atypical antipsychotics (70). This also implies that measurements of D_2_ receptor occupancy with antipsychotics can be affected by the chemistry of the radioligands used (i.e., lipid-soluble spiperone, nemonapride versus water-soluble dopamine, raclopride) (71–73). D_2_ receptor occupancy by atypical antipsychotics such as clozapine and quetiapine will be reduced by (^11^C)raclopride less so than if lipid-soluble radioligands such as (^11^C)methylspiperone were used (63, 73, 74). Therefore, differences in D_2_ receptor occupancy between clozapine, quetiapine, and other antipsychotics could be influenced by the chemistry of the radioligands used (75). This intriguing interpretation, developed using *in vitro* assays, has not been confirmed functionally. Typical and atypical antipsychotics dissociate with similar temporal kinetics in electrophysiological evaluations, suggesting that the reversal of D_2_ receptor antagonism by typical and atypical antipsychotics does not differ markedly (76, 77). These contradictory results point to the possibility that mechanisms other than receptor occupancy may also be involved in the outcomes of these assays, although we cannot dismiss the relevance of ligand binding kinetics at D_2_ receptors for achieving antipsychotic efficacy (24, 38).

## Striatal D_2_ Receptor Density and Blockade in Treatment-Resistant Schizophrenia

**Figure 1:**
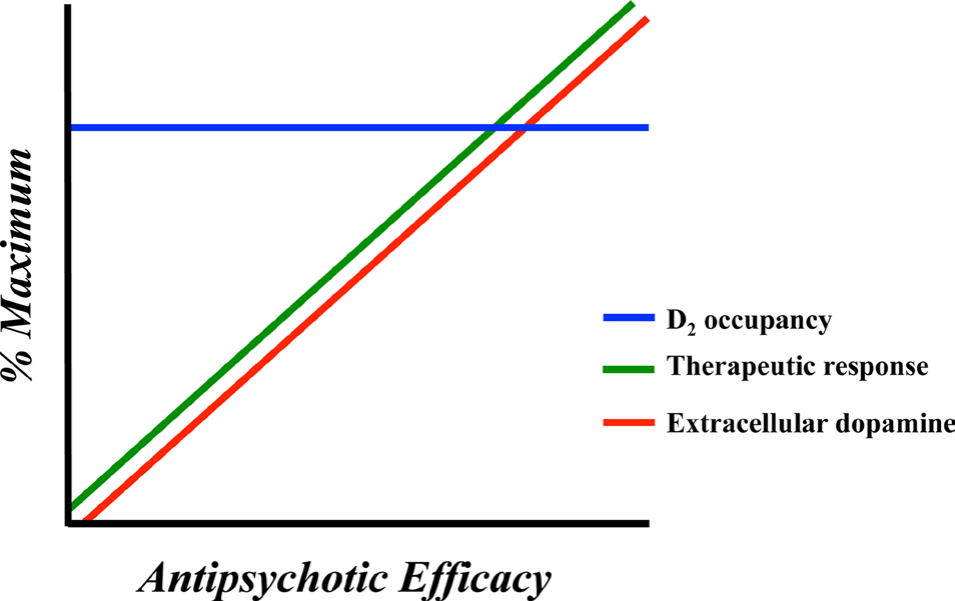
Representation of the neurochemical factors affecting antipsychotic response in humans and animal models. Antipsychotic response is optimal in concert with elevated extracellular dopamine levels. D_2_ receptor occupancy is less dynamic and appears stable during time periods characterized by both therapeutic efficacy and antipsychotic failure.

As already mentioned above, the blockade (or occupancy) of a proportion of D_2_ receptors is not a working antipsychotic mechanism for a significant number of patients with schizophrenia (31). In fact, roughly one-third of individuals with schizophrenia are resistant to treatment with first-line antipsychotics despite sufficient D_2_ receptor occupancy (19). Clozapine, which works at a relatively low (∼40%) striatal D_2_ receptor occupancy (20, 21, 78, 79), is the most effective antipsychotic in the majority of patients refractory to other antipsychotic medications (18, 32, 80). If we hypothetically accept the suggestion that this outcome is not attributable to D_2_ receptor binding kinetics (77), we begin to consider other dopaminergic mechanism that may account for this apparent discrepancy. A growing literature supports the idea that additional dopaminergic mechanisms may underlie therapeutic efficacy of antipsychotic drugs (24, 38). Some patients who respond to first-line antipsychotic treatment experience diminished treatment efficacy over time (23), which can lead to treatment non-compliance and relapse (81). Diminished antipsychotic efficacy may also occur despite stable D_2_ receptor occupancy (82). These dynamics are depicted in **Figure 1**. The opposite has also been observed with long-term antipsychotic efficacy occurring despite decreasing D_2_ receptor occupancy (89–85).

Acquired resistance to antipsychotics (tolerance) could involve antipsychotic-induced dopamine receptor supersensitivity, potentially resulting from D_2_ receptor upregulation, consequent to chronic D_2_ receptor blockade (34, 86, 87). In patients with schizophrenia, antipsychotic-induced dopamine supersensitivity is thought to impair treatment efficacy, promote relapse to psychosis, and also worsen psychotic symptoms (88–90). In laboratory animals, antipsychotic-induced dopamine supersensitivity produces loss of antipsychotic efficacy (35, 91, 92) and an exaggerated behavioral response to dopamine agonists (35, 93–95). However, the link to antipsychotic-induced striatal D_2_ upregulation is complex. Changes in levels of dopamine receptor expression in patients have not been replicated reliably by independent research groups (96, 97). Recent studies using animal models also show tolerance to antipsychotics despite clinically representative levels of striatal D_2_ receptor blockade, as measured either with *in vivo* imaging (38) or *ex vivo* receptor autoradiography (35, 91). Antipsychotic-induced dopamine supersensitivity and tolerance to antipsychotics can also be dissociable from changes in striatal D_2_ receptor density (35). Thus, changes in striatal D_2_ receptor expression are not always predictive of either changes in antipsychotic efficacy or the emergence of antipsychotic-induced dopamine supersensitivity (24, 35, 38, 98, 99), although high doses of antipsychotics may upregulate striatal D_2_ receptors (100).

Beyond changes in striatal D_2_ receptor density, chronic antipsychotic treatment can also increase D_2_ receptor function, and this has been linked to dopamine supersensitivity and acquired antipsychotic tolerance. When D_2_ receptors are coupled to G_i/o_ proteins, they are in a functional, high affinity state for dopamine (referred to as D_2_^HIGH^). When D_2_ receptors are uncoupled to G_i/o_ proteins, they are in a functionally inert, low affinity state for dopamine (D_2_^LOW^). As such, the proportion of D_2_^HIGH^ can modulate dopamine signaling *via* D_2_ receptors. The link between antipsychotic tolerance and changes in striatal D_2_^HIGH^ sites comes largely from work in animal models showing that chronic antipsychotic treatment increases striatal D_2_^HIGH^ levels (35, 91, 101). Antipsychotic treatment regimens that promote behavioral dopamine supersensitivity and antipsychotic treatment tolerance produce an even greater increase in D_2_^HIGH^ sites (91). D_2_^HIGH^ receptor elevation and antipsychotic-induced dopamine supersensitivity also follow a similar time course (35). However, D_2_^HIGH^ sites can increase early in antipsychotic treatment, before any behavioral evidence of dopamine supersensitivity or treatment tolerance (35). In addition, antipsychotic dosing regimens that do not produce dopamine supersensitivity can still increase striatal D_2_^HIGH^ sites (91, 101). Furthermore, there is no conclusive evidence of elevated D_2_^HIGH^ receptors in patients with schizophrenia [see (102)]. Thus, there is likely a link between changes in D_2_^HIGH^ sites and acquired antipsychotic treatment tolerance, but this requires further study.

## Dopamine D_2_ Receptor Isoforms and Schizophrenia

**Figure 2:**
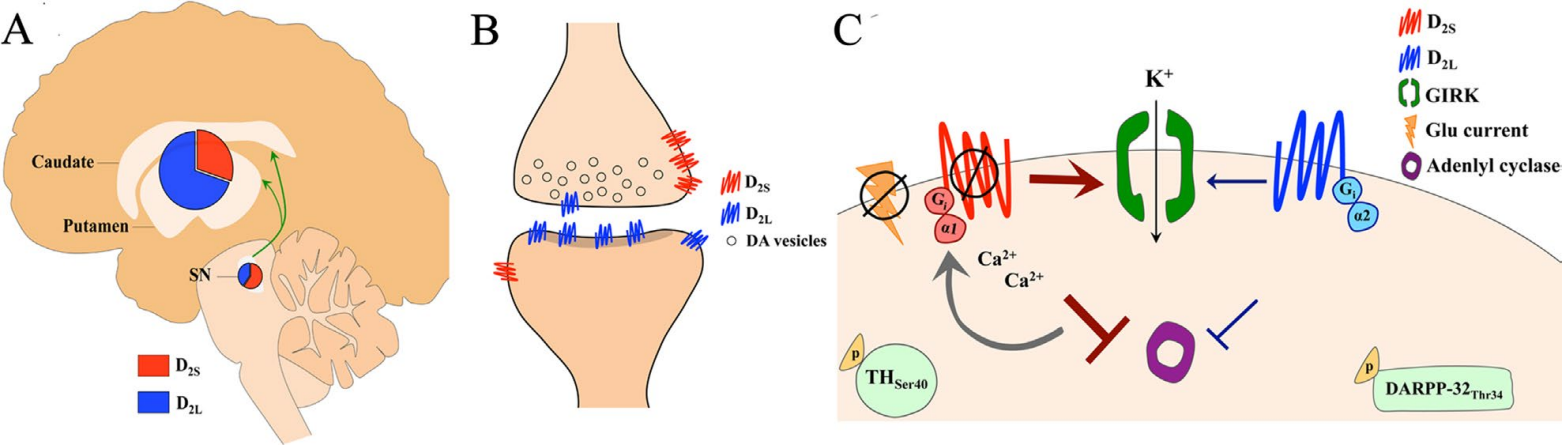
**(A)** Uneven expression of dopamine D_2_ receptor isoforms (short, D_2S_ and long, D_2L_) in the human midbrain (substantia nigra, SN) and striatum (caudate nucleus and putamen). D_2L_ is predominant in the striatum, while D_2S_ is prevalently expressed in the midbrain. This unbalanced D_2L_/D_2S_ ratio is observed across species. **(B)** Schematic of a synaptic contact between a dopaminergic terminal projecting from SN and a somatodendritic spine in the striatum shows the unbalanced D_2L_/D_2S_ ratio on midbrain and striatal neurons. **(C)** Distinct physiological effects are mediated by the two D_2_ receptor isoforms. Both D_2S_ and D_2L_ receptors inhibit adenylyl cyclase, though D_2L_-mediated inhibition is weaker, *via* G_i_α_1_and G_i_α_2_, respectively. D_2S_ stimulation leads to phosphorylation of tyrosine hydroxylase (TH) at serine 40 in nigrostriatal dopaminergic neurons, whereas D_2L_ stimulation leads to phosphorylation of dopamine and cAMP-regulated phosphoprotein of 32 kDa (DARPP-32) at threonine 34, in medium spiny neurons. D_2S_, but not D_2L_, activates G protein-gated inwardly rectifying potassium (GIRK) conductance, which is Ca^2+^ sensitive. D_2S_, but not D_2L_, inhibits excitation in response to glutamate (Glu) currents.

The majority of the cells expressing D_2_ receptors in the striatum are neurons with medium-sized cell bodies and spiny dendrites (medium spiny neurons, MSNs, about 95% of all cells in this region), which are postsynaptic to dopaminergic terminals projecting from the midbrain, among other regions; for an overview, see Refs. (24, 103). The striatum also contains presynaptic D_2_ receptors expressed on dopaminergic axon terminals, which represent only a small percentage of the total D_2_ receptor pool found in the striatum and may have a different molecular structure (104). Accordingly, there are two isoforms of dopamine D_2_ receptors deriving from alternative splicing of exon 6 to produce the long (D_2L_) and the short (D_2S_) forms of the protein (105–107) (**Figure 2A–C**). Both isoforms appear to regulate dopaminergic firing (108), but only D_2S_ controls Ca^2+^-mediated autoinhibition (109, 110). Furthermore, post-synaptic D_2S_, but not D_2L_, controls MSN excitability in rodents (111) and likely in humans (112), despite its pre-dominant presynaptic localization. These effects are likely a consequence of the distinct molecular mechanisms linked to D_2_ receptor isoforms (113–116) (**Figure 2C**).

The expression of D_2_ isoforms in the mammalian brain is distributed unevenly (**Figure 2A**). Genomic studies of human and rodent D_2_ mRNA, which share ∼95–99% homology (117), report that while D_2L_ and D_2S_ mRNA are widely expressed in the brain, D_2L_ mRNA is highly expressed in the striatum (i.e., caudate nucleus and putamen) relative to D_2S_ mRNA (117–120). Investigation of D_2_ protein expression in primates shows that D_2L_ is highly expressed in the striatum and found specifically on MSNs and cholinergic interneurons, while D_2S_ is instead expressed on dopaminergic axons (121). In the cortex and midbrain, D_2L_ is mostly expressed on neuronal somata, while D_2S_ is found on somata, dendrites, and axon terminals (121). Interestingly, high potency antipsychotics (with high affinity for the D_2_ receptor) appear to selectively bind those receptors expressed in the striatum (a structure with high D_2L_/D_2S_ ratio) (122, 123), supporting the notion that antipsychotics could bind both D_2_ isoforms, but that effective antipsychotic doses would bind largely D_2L_ and only a small proportion of total D_2S_ receptors in the brain. Although this possibility is not completely supported from binding studies using cloned D_2_ receptors in cultured cells (124–127), saturation binding studies and *in vivo* studies with ED50 antipsychotics using transgenic mice (i.e., D_2L_ receptor knockout mice) appear to confirm an antipsychotic selectivity for D_2L_ (128–131). Consistent with these observations, humans studies have shown that more effective antipsychotics have higher D_2_ receptor occupancy in the striatum than in the midbrain (SN). (132, 133).

Postmortem studies using brain tissue from patients with schizophrenia that received antipsychotic treatment prior to death demonstrate a significant increase in D_2L_ mRNA in the caudate nucleus (134), arguing in favor of specific adaptations of D_2L_ in response to chronic blockade with antipsychotics. Studies have reported that D_2_ receptor mRNA adaptations with chronic D2 blockade might (135, 136) or might not (137) associate with membrane receptor expression suggesting that post-transcriptional mechanisms might more robustly control D_2_ receptor trafficking (138). Other studies instead demonstrate direct links between gene transcription and D_2_ receptor expression selectively in the striato-pallidal pathway (139). Currently, the precise action of antipsychotics on the D_2_ receptor isoforms is still inconclusive despite strong evidence from these studies with transgenic rodents.

## Dopamine Synthesis, Release, and Uptake

Dopamine levels in schizophrenia are thought to be higher than in healthy individuals especially during psychotic episodes (140) and antipsychotics are intended to reduce this increased dopamine signaling (13). But it is unclear how this could occur when narrowly considering only D_2_ receptor occupancy (24). D_2_ receptors are expressed in the dendrites, somata, and terminals of dopaminergic neurons (autoreceptors) and in postsynaptic neurons (heteroreceptors). Dopamine stimulation of D_2_ autoreceptors at terminals decreases synaptic dopamine release, while stimulation of somatic D_2_ autoreceptors instead decreases the firing activity of these cells (141). Acute application of antipsychotics with high affinity for the D_2_ receptor has been found to increase dopamine release in projection areas (142), and this increase in dopamine is only minimally driven by increased dopamine neuron firing (143, 144), since application of antipsychotics directly onto somatic autoreceptors of midbrain dopamine neurons causes only modest dopamine release (145). Also, postsynaptic D_2_ heteroreceptors can moderately regulate extracellular dopamine in the striatum *via* GABA transmission, especially if autoreceptors are hypofunctional (131). Altogether, these seminal studies suggest that antipsychotics most effectively control dopamine transmission by targeting receptors in terminals found in the striatum. Interestingly, since most of the striatal receptors are heteroreceptors and only modestly control dopamine release, increases or decreases in extracellular dopamine levels (35, 45) are likely mediated by other mechanisms impacted by antipsychotics (38). These regulatory mechanisms include modifications to dopamine synthesis, release, and uptake.

*Synthesis:* Early studies demonstrated that acute antipsychotic treatment increased dopamine synthesis in *in vitro* (146, 147) and *ex vivo* preparations (148) as well as *in vivo* in rodents (149, 150). This was thought to be mediated by direct modification of the enzyme tyrosine hydroxylase (TH) (151, 152). However, later studies could not find changes in dopamine synthesis *in vivo* in human striatum, while comparable doses of antipsychotics appeared to increase dopamine synthesis in animals (153), thus only partially confirming previous work (149). While this discrepancy between rodents and human data was not clarified, a different enzymatic pathway for the synthesis of dopamine (TH vs. aromatic amino acid decarboxylase, AAAD) in rats and humans seemed a plausible explanation (153, 154). The regulation of extracellular dopamine through an autoreceptor-based mechanism of dopamine synthesis using antipsychotics is complex. In fact, studies have shown that decreasing dopamine synthesis has no therapeutic antipsychotic efficacy (155), and though antipsychotic treatment can either increase or decrease dopamine synthesis capacity (DSC, DOPA decarboxylase mediated L-DOPA conversion to dopamine) independently from D_2_ receptor blockade (156), both effects are associated with an improvement of symptomatology (157–159). These contrasting findings may result in part from the very complex molecular machinery that co-regulates DAT, TH, and D_2_ autoreceptors (160–163), making it unlikely that antipsychotic medications will affect this machinery in a predictable manner.

We previously found that TH expression was not changed by effective doses of typical and atypical antipsychotics in animal models (38). However, TH expression increased when antipsychotics were no longer effective, and this was positively correlated with increased DAT expression (38). Interestingly, although TH expression did not change during antipsychotic efficacy, extracellular dopamine increased and vesicular release of dopamine decreased, suggesting that antipsychotics contributed to modulation of extracellular dopamine *via* reduced uptake rather than modified synthesis. Thus, changes in extracellular dopamine levels can be independent from the synthesis rate and may rely more on autoinhibition and uptake (38, 164), and/or a compensatory activity of TH (160).

*Release*: The idea that antipsychotics control dopamine release primarily by D_2_ autoreceptor blockade first emerged with the results of early molecular pharmacology experiments (146, 147, 165–167) showing that antipsychotics revert the inhibitory effects of apomorphine. Subsequent microdialysis (142) and electrophysiological (144) studies supported these early molecular findings. However, most of the results from these early studies have been obtained with limited experimental preparations such as synaptosomes (146, 165, 167) or have involved the use of neurotoxins to destroy post-synaptic neurons in freely moving microdialysis (142), which incurs severe brain lesions. Thus, the significant interaction of antipsychotics with D_2_ autoreceptors found in these early studies should be considered in light of the fact that these manipulations can disrupt the natur al organization of structures within the brain. Therefore, whether therapeutic doses of antipsychotics *in vivo* control dopamine release uniquely through D_2_ autoreceptors is not completely clear (141). Contemporary researchers working when these early studies were conducted acknowledged that this mechanism was only partially plausible (146, 147, 165). Furthermore, the fact that clozapine, which has moderate binding affinity for D_2_ receptors relative to other antipsychotics (78), is as effective as high potency antipsychotics at increasing depolarization by D_2_ autoreceptor blockade (144) likely suggests that mechanisms other than D_2_ autoreceptor antagonism may be involved in the regulation of dopamine output by antipsychotics. One mechanistic possibility is that, at least for atypical antipsychotics, dopamine release is modified by serotonergic mechanisms. But this is unlikely to fully account for antipsychotic-induced dopamine release, since both typical and atypical antipsychotics evoke release of dopamine (38), but typical antipsychotics have much lower affinities at 5-HT receptors compared to second-generation therapeutics [for an overview, see Refs. (168, 169)].

Another possibility as to how antipsychotics regulate striatal dopamine output is through their direct impact on the vesicular exocytosis at active zones linked with Ca^2+^ channels (170, 171). We previously reported that typical and atypical antipsychotics can accumulate in synaptic vesicles of cultured hippocampal neurons through an acidic trapping mechanism and inhibit Na^+^ channels upon release. The inhibition of Na^+^ channels leads to feedback inhibition of Ca^2+^ influx and reduced vesicular dopamine release (171). We tested this mechanism using antipsychotic treatment regimens reflecting clinically relevant outcomes of antipsychotic efficacy and resistance and found that exocytosis-mediated dopamine release was regulated in distinct ways at different points during haloperidol treatment (38). Specifically, haloperidol inhibited dopamine exocytosis in sub-chronic regimens, i.e., ≤6 days and during treatment efficacy, while dopamine exocytosis was enhanced during chronic antipsychotic treatment associated with loss of behavioral efficacy (38). This distinct regulation of vesicular release of dopamine during sub-chronic versus chronic haloperidol might reflect the involvement of two different mechanisms in which K^+^ channels mediate the inhibition of vesicular release, while Na^+^ channels counteract this inhibition (38). Antipsychotics can regulate dopamine release by directly binding the open state of K^+^ channels (i.e. Kv4.3) during depolarization and accelerating the decay rate of inactivation (172–174). This mechanism of action can regulate dopamine release over time independent of depolarization blockade by modifying the intrinsic excitability of dopaminergic neurons (175). Further, changes in K^+^ conductance can shunt the effects of innervating signals onto dopaminergic neurons, preventing changes in dopamine release. One additional mechanism through which antipsychotics may impact dopamine release involves elevation in extracellular dopamine as a consequence of antipsychotic-induced DAT blockade (38), which may activate GIRK currents at axon terminals through an interaction between D2 autoreceptors (24, 38) and Kv1 channels (176). We found that K^+^-mediated release of dopamine is differentially affected during antipsychotic efficacy and failure in freely moving mice undergoing treatment, although it is not yet known if this is due to a direct action of antipsychotics on K^+^ channels or is instead mediated indirectly by elevated endogenous dopamine. Thus, multiple lines of evidence point to the capacity of antipsychotics to impact dopamine release, even though they may not necessarily impact dopamine synthesis.

*Uptake*: In order to appreciate the core mechanism of antipsychotics, it is essential to understand how antipsychotics influence the temporal dynamics of dopamine signaling in the extracellular space within the striatum, the locus of psychosis (9). Data from early studies described above provided copious evidence that antipsychotics block D_2_ receptors and that this is sufficient to restore dysregulated dopamine signaling in many human patients, at least for some period of time. However, these early studies did not distinguish appropriately between antipsychotic action on pre- and post-synaptic D_2_ receptors (141), and it is therefore unclear which D_2_ receptor type accounts for the clinical outcomes generated by antipsychotics (24). Likewise, it is not clear what happens to dopamine released into the extracellular space when antipsychotic drugs prevent its binding to D_2_ receptors (24, 38, 169). Under normal physiological conditions, most extracellular dopamine is recycled by means of re-uptake by DAT and remaining transmitter diffuses away (177). Dopamine re-uptake terminates dopaminergic signaling and prevents toxic consequences of excessive dopamine (178). Accordingly, extracellular dopamine concentration and DAT availability are directly correlated (179). In the absence of DAT-mediated dopamine re-uptake, no other mechanism can maintain homeostatic control of presynaptic function (180), although dopamine spillover also appears to play crucial role in deactivation of dopamine signaling (181). Once dopamine is collected into presynaptic terminals, most of it is recycled and packaged into vesicles (182). The remainder is metabolized enzymatically within the cytosol (180, 183). Therefore, extracellular dopamine concentration is the outcome of dopamine release and clearance (184, 185), and it is of therapeutic relevance to understand how antipsychotics modify this balance (38).

## Antipsychotic Action on DAT

Previous meta-analytical studies have found no consistent evidence for DAT changes in schizophrenia (186), and autoradiographic studies found no antipsychotic-induced changes in DAT density labeled with [^I25^I]RTI-121 ([125I]2 beta-carboxylic acid isopropyl ester-3 beta-(4-iodophenyl)tropane) (187, 188). However, other investigations discussed above report that direct blockade of dopamine uptake contributed to the elevated extracellular dopamine in response to acute antipsychotics (146, 147, 165, 189), although the technology at the time did not allow for a clear distinction between release and uptake kinetics. More recent studies using fast scan voltammetry demonstrated that antipsychotics with high affinity for D_2_ receptors enhanced dopamine half-life by nearly 50% *via* direct DAT blockade and antagonism of D_2_ autoreceptors (190–192). Accordingly, a delayed dopamine half-life results from direct inhibition of DAT, since the decay phase of stimulated dopamine overflow entirely depends on uptake (193). In support of this, striatal slice recordings showed that antipsychotics do not enhance dopamine release after the first stimulation (192), contradicting the idea that D_2_ autoreceptor antagonism by antipsychotics blocks autoinhibition in slices. The direct inhibition of DAT with antipsychotics occurs at low affinity and antipsychotics are less potent than more selective DAT blockers like nomifensine (194–196). This helps us interpret the apparent lack of association between antipsychotics and DAT changes reported by previous studies with low sensitivity methods (187, 188). Since uptake is the main route of elimination of extracellular dopamine (180, 197) and the kinetics of diffusion are independent from release and uptake (177), then DAT blockade by antipsychotics could explain the increase in dopamine and dopamine metabolites observed in previous microdialysis studies (198–202) as well as the prolonged half-life of dopamine stimulated by K^+^ (189).

Additional findings from *ex vivo* studies support a direct interaction between antipsychotics and the DAT. Under normal physiological conditions, increased dopamine release rapidly upregulates DAT membrane expression (203, 204). Effective doses of antipsychotics given sub-chronically (i.e., 2–6 days) inhibits the production of DAT mRNA, but does not alter striatal DAT membrane expression (38). These effects are reversed (i.e., upregulation of DAT mRNA and protein) during chronic antipsychotic treatments associated with loss of behavioral efficacy (38). We and others (205) have found similar DAT adaptations *in vivo* (38). MicroPET imaging using [18F]FP-CMT ([18F] N-3-fluoropropyl-2-beta-carbomethoxy-3-beta-(4’ methylphenyl)) nortropane, with superior properties for imaging the DAT in the living brain (38, 206), was applied to rats at baseline and follow-up (i.e., during loss of antipsychotic efficacy). Rats show an increase in DAT availability (binding potential; BP_ND_) during antipsychotic failure, suggesting the putative relevance of dopamine clearance for achieving antipsychotic therapeutic response, at least in animal models. Interestingly, changes affecting DAT expression and corresponding behavioral responses to antipsychotics are accompanied by a stable and clinically relevant D_2_ receptor blockade (69%) and by increased or decreased extracellular dopamine in the striatum, during the expression of antipsychotic efficacy and failure, respectively (35, 45). Furthermore, the importance of DAT function in antipsychotic efficacy is supported by genetic studies showing an association between clozapine efficacy and DAT gene polymorphism (207). Regarding the question of where dopamine goes when both presynaptic and postsynaptic D_2_ receptors are blocked, these studies suggest that it might be captured by DAT, which is upregulated by clinical doses of antipsychotics (38). However, contrary to the obvious theoretical expectation that reduced dopamine would optimize antipsychotic therapeutic response, we found that it coincided with loss of antipsychotic efficacy. This counterintuitive result has been elaborated elsewhere (24, 38), but it will be briefly recapitulated in the next section and discussed in the context of antipsychotic-resistant schizophrenia.

## Dopamine Autoinhibition as a Feature of Antipsychotic Responsiveness

We have previously proposed a model of antipsychotic efficacy, based on the potential therapeutic properties of endogenous dopamine, by taking into account a number of factors encountered in the clinic and in experimental studies with humans and animals (24, 38). We suggested that antipsychotic efficacy, as observed in animals treated with continuous doses of antipsychotics reaching clinically relevant D_2_ receptor blockade, is driven by dynamic interactions between endogenous dopamine and presynaptic D_2_ receptors. This suggestion is justified by independent but related findings showing that antipsychotic efficacy occurs in conjunction with high striatal extracellular dopamine in humans and animals (35, 38, 45, 46), while only a proportion of the total striatal D_2_ receptors are blocked with antipsychotics in human patients (13, 62) and animals (35, 38). On the other hand, antipsychotic treatment failure is observed when extracellular dopamine (35, 38, 45), but not D_2_ receptor blockade (38), is decreased (**Figure 1**). This fluctuation in extracellular dopamine and antipsychotic response over continuous treatment regimens characterized by stable D_2_ receptor blockade led us to hypothesize that antipsychotics impact the interaction between endogenous dopamine and the D_2_ receptor pool available for binding. Under physiological conditions, spontaneous release of dopamine stimulates a greater proportion of D_2_ than D_1_ receptors (208, 209) and antipsychotics can bind to all dopamine receptors (24, 210). Therefore, when therapeutic doses of antipsychotics reach the brain, about 70% of D_2_ receptors will be blocked along with a modest proportion of D_1_ receptors. As a consequence, endogenous dopamine will interact with spare dopamine receptors and particularly with D_2_ receptors, since this type, relative to D_1_ receptors, is stimulated by low levels of dopamine (209). The resulting neuronal response will be dictated by the molecular characteristics of the D_2_ receptors (i.e., G_i/o_ inhibitory coupled protein). During phasic dopamine release (i.e., that which would be expected to induce a psychotic episode in schizophrenia), dopamine reaches presynaptic autoreceptors, producing antipsychotic-dependent dopamine-mediated autoinhibition and a corresponding antipsychotic efficacy (24, 38).

This autoinhibition might be mediated by the D_2S_ isoform since the two splice variants have distinct functions and are unevenly distributed within the striatonigral dopaminergic circuitry (**Figure 2A, C**). Furthermore, antipsychotics appear to preferentially bind dopamine receptors in the striatum (123), a brain structure with predominant expression of D_2L_ as discussed above, and dopamine exhibits higher binding affinity for D_2S_ in transgenic mice (130) and in cell culture (113). Together these data suggest that therapeutic doses of antipsychotics in the brain cause a functional segregation of D_2S_ and D_2L_, which based on the data available until now could overlap with a functional segregation of pre- and post-synaptic D_2_ receptors (**Figure 2A, C**). It should be noted that both isoforms are expressed in pre- and post-synaptic neurons and the functional segregation might also occur within the same cells (**Figure 2B**). In support of this theory are studies with human schizophrenia patients demonstrating selective reduction in expression of D_2S_ mRNA (211), potentially indicative of a desensitization of the short isoform in response to increased dopamine activity on this receptor. On the other hand, postmortem studies also show that D_2L_ mRNA is upregulated in patients with schizophrenia (212), which may indicate an adaptive response to chronic blockade (119).

**Figure 3:**
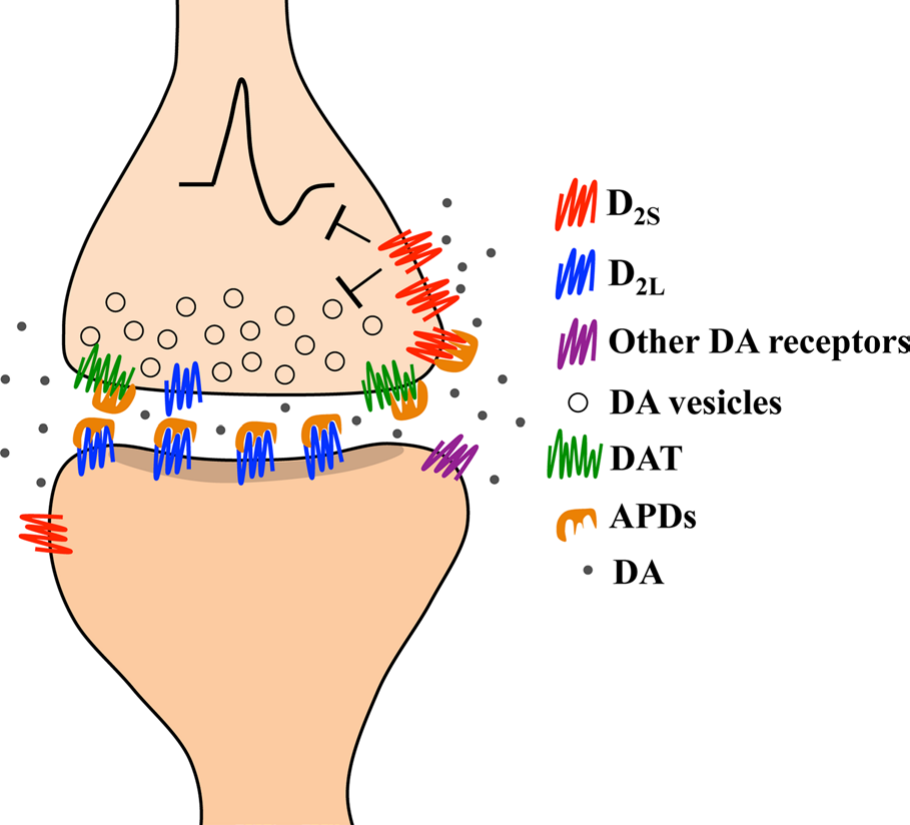
Representation of the hypothesized pharmacological mechanism underlying a therapeutic response in schizophrenia based on human and animal studies. Therapeutic doses of antipsychotic drugs (APDs) block about 70% of striatal D_2_ receptors. APDs mostly block heteroreceptors, which are more often D_2L_ than D_2S_, as well as a smaller proportion of autoreceptors (which are more often D_2S_ than D_2L_). APDs also block the dopamine transporter (DAT). The combined blockade of D_2L_ heteroreceptors and DAT causes synaptic accumulation of dopamine that allows stimulation of spare D_2S_ receptors. Phasic release of dopamine in response to environmental changes will trigger an enduring autoinhibition since extracellular dopamine levels are already elevated. We hypothesize that the autoinhibition triggered by a phasic discharge of dopamine during antipsychotic treatment is the mechanism underlying a therapeutic antipsychotic response.

Since phasic discharge leads to large extracellular increases in dopamine (213) and is thought to underlie psychotic experiences (9, 41, 46, 140, 214–217), we propose that a therapeutic antipsychotic response is obtained by antipsychotic drugs when an adequate proportion of D_2_ receptors is blocked and extracellular dopamine levels are sufficiently elevated to trigger autoinhibition. This crucial combination of effects is achieved by the direct blockade of DAT by antipsychotics (38, 146, 147, 165, 189), which allows for an accumulation of synaptic dopamine that reduces the threshold at which phasic dopamine activates homeostatic autoinhibition. The antipsychotic-induced facilitation of dopamine autoinhibition, mediated by DAT blockade and D_2_ autoreceptor stimulation, which may serve as an antipsychotic mechanism is depicted in **Figure 3**. Although we have arrived at this hypothesis by analyzing multiple experimental observations, which sometimes lack corresponding human studies, our functional predictions on the association between extracellular dopamine and antipsychotic therapeutic responsiveness in humans and animals have been observed by a number of independent groups (35, 38, 45–48, 49, 218). In the following section, we will provide naturalistic examples of the potential importance of functional DAT to understanding antipsychotic-resistant schizophrenia.

## The Role of DAT in Antipsychotic-Resistant Schizophrenia: Lessons from Aging and Drug Addiction

**Figure 4:**
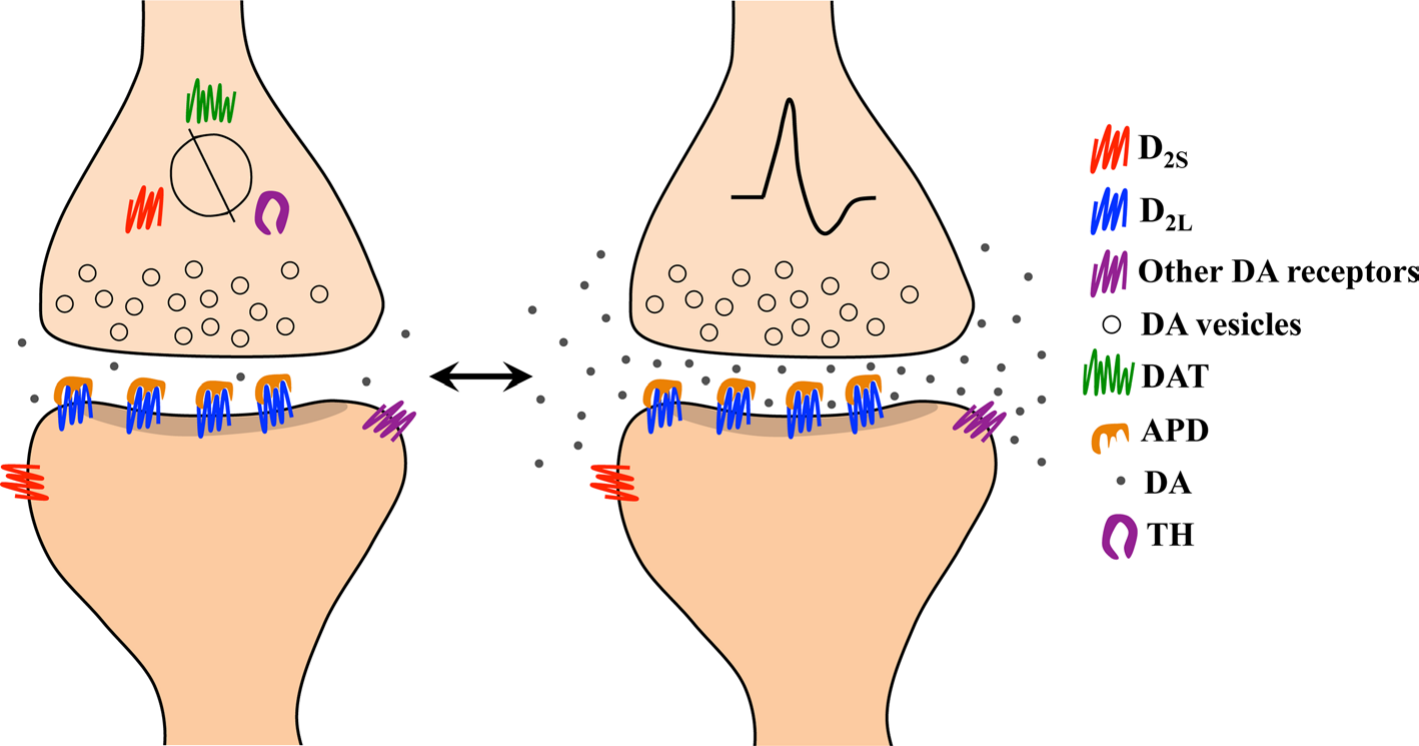
Representation of the pharmacological mechanism underlying the absence of therapeutic response in antipsychotic-resistant schizophrenia based on our model. (Left) Aging and/or addictive drugs consumed before antipsychotic treatment begins (i.e., in first episode psychosis) lead to reduced expression of the dopamine transporter (DAT), D_2_ autoreceptors, and tyrosine hydroxylase (TH), as these proteins appear to be co-regulated, at least in rodents. (Right) During environmentally evoked phasic dopamine release, impaired capacity for autoinhibition results from low levels of DAT and D_2_ autoreceptors. The resulting post-synaptic stimulation contributes to psychosis despite a significant blockade of D_2_ receptors by antipsychotic drugs (APDs).

If extracellular dopamine levels contribute to the generation of a therapeutic antipsychotic response and DAT is the main physiological regulator of extracellular dopamine levels, then DAT should have a role in the expression of antipsychotic-resistant schizophrenia. Furthermore, if DAT activity quickly adapts to changes in extracellular dopamine, then it would be surprising if DAT was unaltered in schizophrenia, a disorder with symptoms attributed to dysregulated dopamine neurotransmission. We have described above how blockade of DAT may be a critical factor in antipsychotic efficacy, since DAT blockade allows accumulation of extracellular dopamine and consequently dopamine-mediated autoinhibition upon phasic transmitter release. We have also described research showing that antipsychotics given to rodents at therapeutic doses induce DAT upregulation during loss of behavioral efficacy (38). The loss of efficacy in this scenario coincides with a reduction in extracellular dopamine, which we predict reduces the capacity of dopaminergic terminals to undergo autoinhibition upon phasic release. On the other hand, we introduce below an additional scenario in which reduced expression of DAT may also prove deleterious in terms of antipsychotic therapeutic efficacy. Although theoretically low DAT expression would allow accumulation of extracellular dopamine, which we hypothesize is essential for therapeutic efficacy (**Figure 3**), proteins regulating extracellular dopamine levels including DAT, D_2_ autoreceptors, ion channels, and dopamine synthesis machinery appear to be co-regulated (131, 160–163, 172). Thus, DAT downregulation at the expression level may also negatively impact the capacity of dopaminergic terminals to undergo autoinhibition. We predict that downregulation of proteins regulating physiological dopamine neurotransmission at baseline (i.e., tonic neurotransmission) could be the underlying neurobiology of primary antipsychotic treatment-resistant schizophrenia. **Figure 4** depicts a scenario in which the absence of autoinhibition due to ablated DAT expression and autoreceptor co-regulation allows for an enduring stimulation of free unbound post-synaptic receptors, leading to psychosis despite a reduction in dopamine release overall. We can characterize this condition as a form of dopamine supersensitivity driven entirely by presynaptic adaptations. Although DAT expression has been found to change in animal models of antipsychotic responsivity, it cannot be assumed that the same mechanism applies in humans with schizophrenia. Indeed, data may differ across species as already shown with D_2_ receptor binding (219) and dopamine synthesis (153). Therefore, why should this principle of species incompatibility not also apply for dopamine uptake? We can gain a better understanding of this issue only after testing it in human patients.

### Aging

Meta-analytical studies report that DAT levels in schizophrenia are mostly decreased, unchanged, and sometimes increased (186). These data were obtained mostly with untreated patients and therefore we hypothesize (24, 38) that the variability of these results was consequent to genetic factors (220–222) and age. For example, DAT density can decrease with age (223). Based on our proposal, reduced DAT expression as a result of aging can decrease autoinhibition mediated by antipsychotics, due to the co-regulation of autoreceptors described above, and thus reduce antipsychotic response. Interestingly, many of the patients that participated in the aforementioned study were ∼40 years old, the age associated with a decline in DAT density (186). It would have been of interest to administer antipsychotics to these individuals and measure their responsiveness. Perhaps, they would have been non-responsive or less responsive than younger individuals and/or those with higher DAT availability. However, these were not the aims of those studies. In support of this suggestion, a previous study (19) showed that the average age of patients with treatment-resistant schizophrenia was 42 years old, while patients responsive to treatment were 25 on average. Interestingly, the treatment with antipsychotics yielded similar levels of D_2_ receptor occupancy (19).

Aging is an important factor underlying neuropharmacological responsiveness mediated by the dopaminergic system, since D_2_ receptors and DAT expression decline naturally in healthy aging individuals (224–229). The reduction in D_2_ receptor and DAT expression is unrelated to dopamine neuron loss (229) and has profound consequences on the antipsychotic therapeutic dosing required to obtain therapeutic responsiveness in schizophrenia (230). Aging can also reveal genetic predisposition to suboptimal DAT and D_2_ receptor functions affecting cognitive performance in healthy individuals (231), and it can trigger degeneration of dopaminergic neurons through increased nitrative damage resulting from excess cytosolic dopamine due to an imbalance in DAT/VMAT (vesicular monoamine transporter-2) expression (232). This form of toxicity, deriving from an excess of cytosolic dopamine, has relevance to understand some of the extrapyramidal symptoms (232) and the loss of brain tissue in patients with schizophrenia (233). Although it is not clear if DAT changes are a main player in maladaptive functional and structural changes, both are often observed in schizophrenia and might affect antipsychotic response in elderly patients with schizophrenia (234, 235). While aging could explain the expression of antipsychotic treatment resistance in older patients, it is not yet clear why DAT function would affect antipsychotic responsiveness in younger individual with schizophrenia. A theoretical suggestion is provided in the following section.

### Drug Addiction in Schizophrenia

Epidemiological studies report that nearly half of patients with schizophrenia also suffer from drug addiction (236, 237). This is about four times more prevalent than in the general population (238). If we consider that the recreational consumption of addictive drugs is common in the general population (i.e., 84% for alcohol consumption), but only a small proportion of individuals exposed to drugs of abuse become drug addicted (239, 240) and that this happens about four times more often in patients with schizophrenia, then it is possible that many of the remaining ∼50% of patients with schizophrenia without formal diagnosis for drug addiction likely consume at least some class of addictive drugs as well. The most commonly consumed drugs in patients with schizophrenia include alcohol, psychostimulants, cannabis, and tobacco (236–238). It has been suggested that patients with schizophrenia may use illicit substances to self-medicate their symptoms (236, 238, 241) as well as the side effects of antipsychotic medications (242), as self-medication with addictive drugs is indeed common in patients with mental illness (243).

All addictive drugs impact the dopaminergic system in the midbrain and in striatal structures (244, 245), a main component of the brain reward circuitry (246), and likely will also impact the DAT (221, 247–253). We theorize that consumption of substances of abuse to medicate pre-psychotic symptoms during the prodromal period is very likely to trigger psychotic episodes, and importantly, to weaken (or blunt) antipsychotic response since repeated exposure to addictive substances (including psychostimulants, cannabis, tobacco, alcohol and heroin) can decrease DAT membrane expression (248–253). This suggestion is based on our model describing the importance of functional DAT to facilitate antipsychotic mediated autoinhibition (**Figure 3**).

Although reduced DAT expression might be assumed to promote the effectiveness of antipsychotics, since uptake blockade with antipsychotics results in synaptic accumulation of dopamine and facilitates autoinhibition upon phasic dopamine release, receptor desensitization due to a corresponding downregulation (or phosphorylation) of autoreceptors may prevent the occurrence (or reduce the likelihood) of autoinhibition altogether (**Figure 4**). Not only are the DAT and D_2_ autoreceptors co-regulated, along with ion channels and the dopamine synthesis machinery (131, 160–163, 172), but reduced DAT, reduced D_2_ receptor expression, and reduced dopamine release can all be found in human psychostimulant users (254) and are linked to blunted striatal dopaminergic transmission in human patients with co-morbid schizophrenia and drug addiction (255).

It should be noted that the mechanisms described here and depicted in **Figure 4** apply to the primary form of antipsychotic-resistant schizophrenia and not to acquired antipsychotic resistance (i.e., tolerance) observed in humans (23) and in animal models (35, 38, 45, 91). This distinction is fundamental since DAT plasticity underlying the acquired resistance to antipsychotics is different than what is described here. In fact, based on our own findings from animal models, chronic antipsychotic treatment up-regulates DAT (38), while other studies with humans and animals show that repeated exposure to addictive drugs reduce DAT (254, 256) and both conditions can lead to lack of antipsychotic response [see Ref. (38) for an expanded discussion of acquired antipsychotic resistance and **Figure 4** for a depiction of primary resistance]. The description of several forms of DAT plasticity induced by psychotropic drugs is beyond the scope of this paper, but it should be acknowledged that the reduction of DAT expression with chronic addictive drug use is not absolute and is sensitive to several factors including treatment regimen, drug class, among others, as summarized in these interesting studies (257–259).

### Antipsychotic-Resistant Schizophrenia: A Hypothetical Example

A young person who may not be aware of an underlying genetic predisposition to psychosis who becomes exposed to substances of abuse at the same rate as other non-predisposed individuals may risk impacting his or her capacity to buffer excess extracellular dopamine *via* drug-induced downregulation of DAT expression. This individual may seek medical intervention upon first experience of psychosis, at which time he or she will receive antipsychotic treatment and may already face reduced therapeutic efficacy due to the drug-related changes in DAT expression. On the other hand, if patients have no history of addictive substance consumption before starting antipsychotic treatment and begin using moderate doses of addictive drugs thereafter, we speculate that the effects of antipsychotics and certain categories of addictive substances on the expression and function of the dopaminergic machinery (DAT, TH, D_2_ receptors) may counterbalance one another (24), producing some therapeutic efficacy for a period of time. This might explain the high rate of smoking and use of illicit substances among patients with schizophrenia.

In summary, we propose that antipsychotic efficacy in patients with schizophrenia and particularly the contribution of DAT expression to antipsychotic response may be influenced by genetic factors as well as environmental factors such as age or history of drug use/abuse. We hypothesize that a history of drug use prior to onset of schizophrenia could be a potential risk factor to becoming antipsychotic treatment resistant, since previous exposure to addictive substances may decrease DAT expression and impair the synaptic machinery required for autoinhibition, which we theorize underlies antipsychotic responsiveness during medical intervention in schizophrenia. Antipsychotic-resistant schizophrenia patients may still respond to clozapine despite reduced DAT expression, because clozapine in particular stimulates serotonin release [for an overview, see Refs. (168, 169)], which suppresses dopaminergic firing (259–262) and thus may compensate for the absence of dopamine-mediated autoinhibition. Though based on a breadth of clinical and bench research, this theoretical suggestion is speculative and requires validation. A more thorough evaluation of this possibility might entail assessment of patient demographics, including history of drug use or abuse, as well as the drug classes used and frequency of use, along with a history of therapeutic responsiveness or resistance when treated with typical or atypical antipsychotics.

## Conclusion

Although we acknowledge the genetic and neurobiological complexity of schizophrenia and its relevance for the efficacy of pharmacological treatment, we propose that sufficient DAT expression in the brains of patients with schizophrenia may be necessary for an adequate antipsychotic response in first episode psychosis. Particularly, we suggest that the antipsychotic-mediated reduction in dopamine re-uptake by direct DAT blockade allows accumulation of dopamine in the synaptic cleft, which increases the efficiency by which phasically discharged dopamine triggers presynaptic autoinhibition. Furthermore, given the apparent selectivity of antipsychotics for the D_2L_ isoform and the predominant presynaptic expression of D_2S_ in the midbrain, phasic dopamine is likely to activate D_2S_, which specifically reduces neuronal excitability. Thus, the functional and spatial segregation of the D_2_ receptor isoforms within the striatum and midbrain may contribute to the generation of an antipsychotic response. We further propose that consumption of addictive drugs prior to onset of schizophrenia symptoms might reduce expression of both DAT and D_2_ autoreceptors and will increase the risk of antipsychotic resistance upon treatment. Similarly, since DAT and D_2_ receptor expression decline with age, aging itself may serve as a risk factor for antipsychotic resistance. Although these hypotheses require further validation, our theory points to the importance of a functional level of membrane DAT expression in patients with schizophrenia in order to gain therapeutic benefit from antipsychotics.

## Author Contributions

DA conceptualized the ideas presented and wrote the first draft. DA, AK, A-NS, and AH wrote the final manuscript. AK made the figures. All authors have read and approved the final version of the manuscript.

## Funding

DA is supported by the Deutsche Forschungsgemeinschaft (AM 488/1-1) and by the Brain & Behavior Research Foundation (NARSAD Young Investigator Award 2018). AK is supported by the National Institutes of Health (DA044782). A-NS is supported by a salary award from the Fonds de la Recherche du Québec-Santé (28988).

## Conflict of Interest Statement

A-NS is a consultant for H. Lundbeck A/S. This had no influence on the manuscript’s content. The remaining authors declare that the research was conducted in the absence of any commercial or financial relationships that could be construed as a potential conflict of interest.
